# Potential of Using Cell-Free DNA and miRNA in Breast Milk to Screen Early Breast Cancer

**DOI:** 10.1155/2020/8126176

**Published:** 2020-07-02

**Authors:** Qinghe Song, Yingying Zhang, Hongtao Liu, Yuguang Du

**Affiliations:** ^1^State Key Laboratory of Biochemical Engineering and Key Laboratory of Biopharmaceutical Production & Formulation Engineering, PLA, Institute of Process Engineering, Chinese Academy of Sciences, Beijing 100190, China; ^2^Beijing Key Laboratory of Rehabilitation Technical Aids for Old-Age Disability, National Research Center for Rehabilitation Technical Aids, Beijing 100176, China; ^3^Hubei University of Chinese Medicine, Wuhan 430065, China

## Abstract

**Objective:**

An ideal sample source is critical for more reliable and sensitive early detection of nucleic acid changes associated with breast cancer. Breast milk (BM) is a good noninvasive origin for genetic testing of early breast cancer, but cells in BM are easily disintegrated. So we investigate here whether cell-free nucleic acid (cfNA) exists in BM in a more stable form and whether the quality of BM cfNA is good enough for genetic testing.

**Methods:**

A self-designed qRT-PCR method was used to measure the existence and abundance of cfDNA. Quality of cfDNA and cfRNA were detected by capillary electrophoresis. Whole genome bisulfite sequencing and miRNA sequencing were used to explore the sources of cfDNA and cell-free miRNA in BM. The copy number analysis and *z*-test based on whole genome sequencing data were used to determine the integrity of genetic information in BM cfNA.

**Results:**

We found that cell-free DNA and miRNA exist in the studied breast milk samples in a stable form that can tolerate incubation of BM at room temperature for at least 7 days. These cell-free nucleic acids come mainly from breast-derived cells and contain genetic information as good integrity as in BM cells. We further listed some candidate miRNAs as potential biomarkers for research of early breast cancer screening by analysis of previous reports and our data.

**Conclusions:**

Our results suggest that cfDNA and cell-free miRNA in BM might be new noninvasive sample sources for finding early alterations of nucleic acid associated with the initiation and progression of breast cancer.

## 1. Introduction

Breast cancer is one of the commonly diagnosed diseases and is the leading cause of all cancer death in women worldwide. Its incidence under 50 years old is on the rise, particularly in developing countries [1, 2]. In China, the incidence of breast cancer is the highest among female malignant tumors and increases rapidly with age after 20 years old [3, 4].

Screening and early diagnosis for breast cancer is considered effective for reducing breast cancer-related mortalities. Yet mammography, the traditional screening method, is less sensitive on very early breast cancer and not recommended as a routine method for women younger than 50 years old [5]. The younger trend of onset makes reliable early screening for young women an urgent need. At the same time, it is of primary importance to find an ideal noninvasive sample source suitable for routine screening.

During the past decade, increasing attention has been paid to detecting cell-free nucleic acid (cfNA) in plasma, including cell-free DNA (cfDNA) and microRNA (miRNA), which are considered useful for early detection of cancer [6, 7]. However, the cfNA derived from tumor cells, also known as circulating tumor nucleic acid (ctNA), is usually variable and only represents a small fraction of total cfNA [8, 9], especially at the beginning of cancer [10], so that the reliability and sensitivity are the major barriers to the usage of plasma cfNA on early screening for breast cancer.

Breast milk (BM) is a natural noninvasive sample source that has a potential in the study of breast cancer risk. Lactocytes and epithelial cells in fresh BM may offer a noninvasive origin of nucleic acid for examining genetic, epigenetic, or gene expression changes associated with breast cancer [11, 12]. But we found that cells in BM are not stable enough; genomic DNA (gDNA) in BM cells would be completely degraded if BM samples were stored at room temperature (RT) for 48 hours, which is a big obstacle to mass screening due to the unstability of long-distance sample transportation.

In this study, we found that abundant and high-quality cfDNA and cell-free miRNA exist in BM in a relatively stable form at RT. Our results show that cfNAs in BM mainly come from breast-derived cells, including lactocytes (milk secretory cells) and myoepithelial cells (from the ducts and alveoli of the mammary gland), and contain genetic information similar to that in BM cells. Perhaps, these characteristics might make BM cfDNA and cell-free miRNA new noninvasive sample sources for screening early alterations of nucleic acid associated with the initiation and progression of breast cancer.

## 2. Materials and Methods

### 2.1. Human Breast Milk Sample Collection

Human breast milk samples from healthy donors were obtained with informed consent under institutional review board-approved protocols. Samples were derived from local healthy donors from Beijing. Details of sample collection and processing are provided in Supplemental Text (available here).

### 2.2. Nucleic Acid Extraction Procedures

Extraction of cellular RNA was via the traditional TRIzol (Ambion) method, and extraction of genomic DNA (gDNA) was via the QIAamp DNA mini kit (Qiagen). BM cfDNA was extracted using a cfDNA purification kit (SummerBio). BM cfRNA isolation was carried out using a cfRNA purification kit (SummerBio). All nucleic acid extraction processes were carried out following the manufacturer's procedure.

### 2.3. Measurement of cfDNA Abundance in BM

BM cfDNA abundance was measured by using a self-designed qRT-PCR method. Full details of the experiments and result processing are provided in Supplemental Text.

### 2.4. Detection of cfDNA and cfRNA Quality

cfDNA and cfRNA quality was detected by capillary electrophoresis using Qsep100 Bio-Fragment Analyzer (BiOptic) and Agilent 2100 Bioanalyzer, respectively.

### 2.5. Measurement of the RNA Difference between BM Cellular RNA and BM cfRNA by Using RT-qPCR Assays

RNA samples from different donors were subjected to RT-qPCR assays to determine the difference of expression levels of LALBA, K-CSN, B-CSN, ELF5, and TACSTD2, and PPBP, CD99, ITGB3, RGS18, and CD45. Forward and reverse primers for qPCR are listed in Supplemental Table S1. Full details of the experiments are provided in Supplemental Text.

### 2.6. Next-Generation Sequencing (NGS) and Data Analysis

miRNA sequencing and whole genome bisulfite sequencing (WGBS) were performed by LC Sciences (Houston, TX, USA). Details of library generation and NGS data analysis are provided in Supplemental Text. The whole genome sequencing (WGS) library of cfDNA was generated by using a NEB Next Ultra DNA Library Prep Kit for Illumina (NEB) according to the manufacturer's recommended protocol. Processes of quality control, sequencing, and data analysis are provided in Supplemental Text in detail.

### 2.7. Statistical Analysis

Statistical information is indicated in the text or figure legends.

## 3. Results

### 3.1. Abundant and High-Quality cfDNA Exists in BM in a Remarkable Stable Form at RT

**Figure 1:**
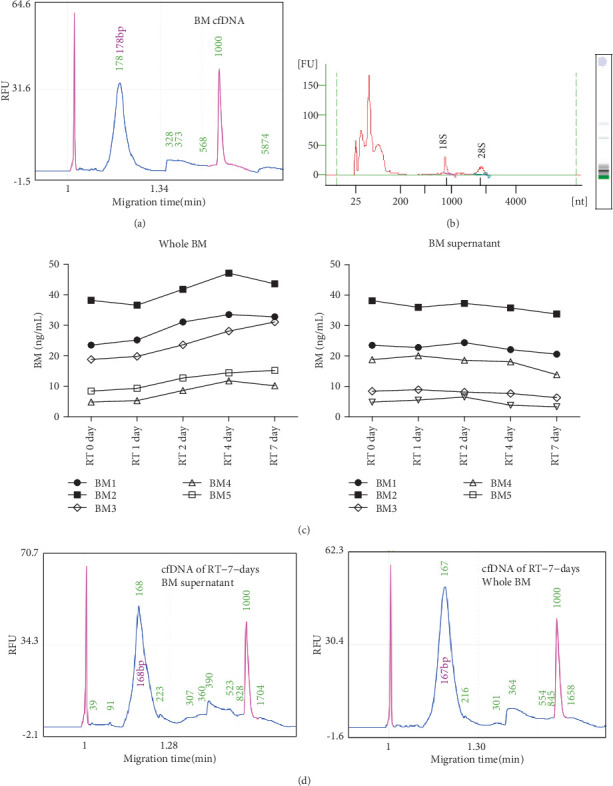
Identification of the existence and stability of cfDNA in human BM. (a) cfDNAs isolated from BM (from donor 1) were detected by Qsep100 Bio-Fragment Analyzer (BiOptic). (b) cfRNAs extracted from fresh BM (from donor 1) were detected by Agilent 2100 Bioanalyzer. (c) The whole BM and BM supernatant from donors 1, 2, 3, 4, and 5 were incubated at room temperature for the indicated time; then, cfDNAs were extracted and detected quantitatively by qRT-PCR; normalized results were showed here. (d) Capillary electrophoresis detection of cfDNA isolated from the whole BM and BM supernatant (from donor 1) after incubation at room temperature for 7 days.

Fresh breast milk (BM) contains cells that may offer a noninvasive source for genetic testing [11, 13]. But our data shows that the genomic DNA (gDNA) in BM cells is almost completely degraded after BM samples were stored at room temperature (RT) for 48 hours (Supplemental Figure S1), such stability is difficult to meet due to the long-distance transportation of samples. At the same time, we found that BM also contains cell-free DNA (cfDNA) and cell-free RNA (cfRNA) (Figures 1(a) and 1(b)), and cfDNA in BM has a major size of ~180 bp fragments (Figure 1(a)) like in plasma. So we wonder if cfDNA is more stable than cellular DNA in BM. To investigate the stability of cfNAs in BM, BM samples from 5 healthy donors were collected; then, the whole BM and isolated BM supernatant from donors were incubated at RT for different time points; then, the abundance of cfDNA was detected. Results show that, after 7 days of RT incubation, the abundance and the major fragment size of BM cfDNA are still good enough (Figures 1(c) and 1(d)). We noticed that, starting from the 2nd day of RT incubation, cfDNA levels showed an upward trend in whole BM samples but a slight downward trend in BM supernatant samples (Figure 1(c)), which strongly suggests that the degraded gDNA of BM cells could have been released into breast milk to become cfDNA. Results of capillary electrophoresis show that BM cfRNA is largely of low molecular weight, and after incubation at RT for 7 days, the fraction of macromolecular cfRNA decreases, but micromolecular cfRNA still maintains abundant (Supplemental Figure S2). These results indicate that abundant cfDNA and micromolecular cfRNA exist in BM in a stable form at RT.

### 3.2. BM cfNA Is Mainly Originally from Breast-Derived Cells

**Figure 2:**
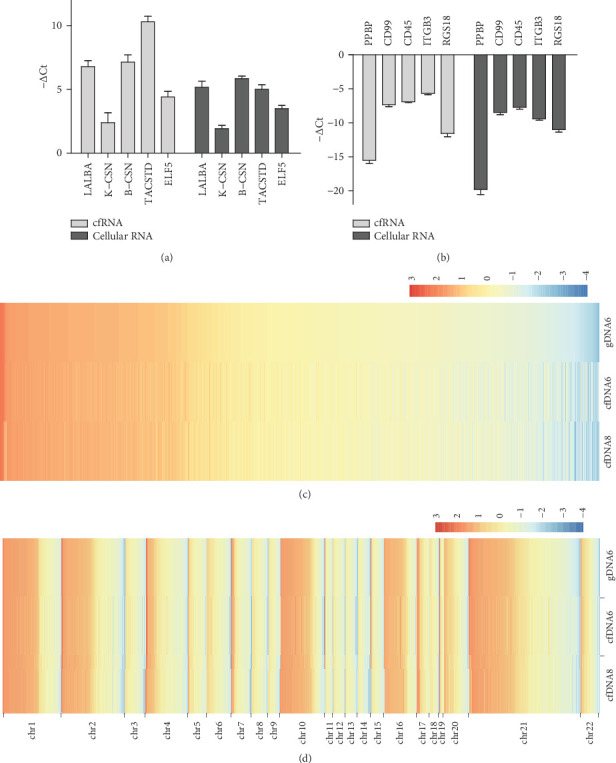
Identification of BM cfRNA deriving mainly from breast-derived cells. RNAs of BM cfRNAs and BM cellular RNA from three different donors (donors 7, 8, and 9) were subjected to RT-PCR to detect mRNA levels of (a) highly expressed genes in breast-related and (b) highly expressed genes in blood-related cells. The graphs show normalized −ΔCt values for the indicated genes (described in detail in Supplemental Text). All error bars denote SEM, *n* = 3. (c) The heat map of methylation levels in every identical reliable slide window of different samples. WGBS data was analyzed with default parameters (1000 bp slide windows, 500 bp overlap). For accuracy, only slide windows with more than 3000 total C reads (methylated and unmethylated) were regarded as reliable for comparison between samples. The graph was drawn with Log2 methylated percentages after mean subtraction and standardization and arranged in descending order across all chromosomes of the gDNA6 sample. (d) The chromosome-separated heat map of methylation levels in identical reliable slide windows of different samples. The graph was drawn with Log2 methylated percentages after mean subtraction and standardization and arranged in descending order in each chromosome of the gDNA6 sample.

Previous reports reveal that the population of BM cells was composed of breast-derived cells and blood-derived cells, and breast-derived cells, including lactocytes (milk secretory cells) and myoepithelial cells (from the ducts and alveoli of the mammary gland), make up the majority of the population of BM cells [14, 15]. In order to determine whether BM cfNAs also mainly come from breast-derived cells, highly expressed genes in breast-related LALBA, K-CSN, B-CSN, ELF5, and TACSTD2 and highly expressed genes in blood-related cells PPBP, CD99, ITGB3, RGS18, and CD45 were chosen, and the expression pattern of these genes in BM cfRNA and BM cellular RNA from different donors was detected using RT-PCR. As shown in Figures 2(a) and 2(b), BM cfRNA exhibits a very high consistency of the expression pattern with BM cellular RNA, having much higher expression levels in breast-related genes than in blood-related genes. These results suggest that BM cfRNA comes mainly from breast-derived cells and shows high similarity between different individuals.

To investigate whether BM cfDNA is mainly derived from breast-derived cells, whole genome bisulfite sequencing (WGBS) was carried out to compare epigenetic similarity between gDNA of BM cells (from donor 6) and BM cfDNA (from donors 6 and 8). The slide window method was applied for methylation level analysis. With window size = 1000 bp and step size = 500 bp, the sum of methylated and unmethylated C read counts were calculated in each window. The methylation level (ML) for C sites in each window is defined as ML = mC reads/(mC reads + C reads). To eliminate the unreliability of data with low sequencing depth, slide windows with total C read counts less than 3000 were filtered. Results show that the methylation levels in different regions of each chromosome are highly consistent between samples (Figures 2(c) and 2(d)). Pearson correlation analysis also suggests high similarity between cfDNA and gDNA (Supplemental Figure S3a). These data suggest that, like BM cells, BM cfDNA comes mainly from breast-derived cells and possesses remarkable epigenetic similarity between different individuals.

### 3.3. Genetic Information of BM cfDNA Is Highly Uniform with the Whole Genome

**Figure 3:**
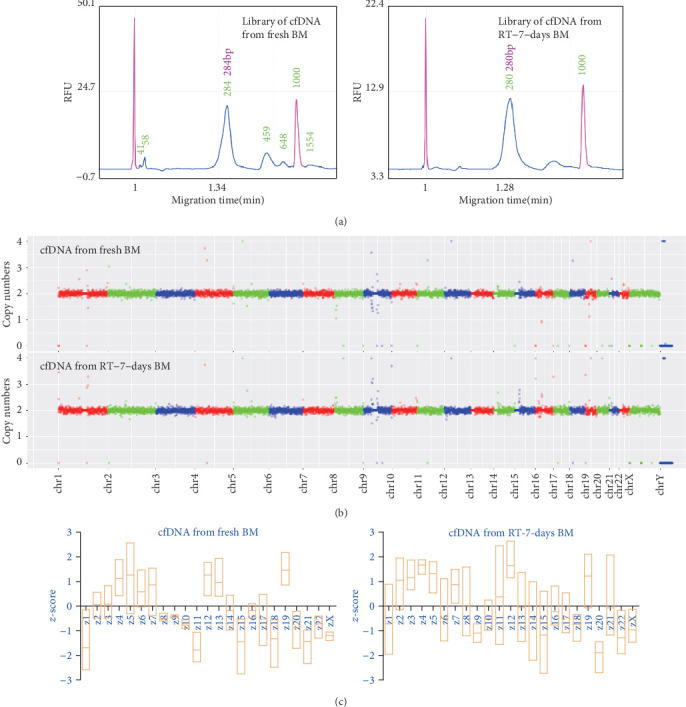
BM cfDNA contains complete genetic information that is highly uniform with the whole genome. (a) Whole genome sequencing (WGS) libraries were generated using cfDNA from fresh and RT-7-days BM (donor 1), then detected by capillary electrophoresis. Results show that the major molecular weight size of three libraries is very similar. (b) WGS data of libraries in (a) were used to analyze copy numbers on a basic unit of 0.5 Mb (described in detail in Supplemental Text). Then, data visualization of copy numbers was plotted by using ggplot2 software. (c) The *z*-score of each chromosome was calculated (described in detail in Supplemental Text) based on WGS data of cfDNAs from three fresh BM and three RT-7-days BM (donors 1, 2, and 3). The graphs show the statistical chromosomal *z*-score of different samples.

We know that plasma cfDNA has a high uniformity with whole genomic DNA. The relative fragment proportion of plasma cfDNA among different chromosomes is very consistent with that of the complete genome, so that prenatal aneuploid testing can be developed via NGS of plasma cfDNA [16]. Our data shows that BM cfDNAs also have these characteristics. Copy number analysis based on whole genome sequencing (WGS) data demonstrates that genetic information of cfDNA from both the fresh BM and RT-7-days BM samples have consistent good integrity on a basic unit of 0.5 Mb (Figure 3(b)). The major molecular weight sizes of libraries from the fresh and RT-7-days BM samples are similar (Figure 3(a)). The *z*-test is a method used to analyze the aneuploid based on WGS data. By this method, a *z*-score is calculated to measure deviation of a chromosomal dosage from a set of euploid control samples [17, 18]. To further testify the uniformity and integrity of BM cfDNA, the *z*-test was utilize to analyzed WGS data of cfDNAs from the fresh and RT-7-days BM samples. Results show that, no matter which cfDNA, the *z*-score of each chromosome is within the normal diploid range (between -3 and 3) (Figure 3(c)). All these data suggest that, like plasma cfDNA, BM cfDNA contains well-distributed genetic information that is highly uniform with the whole genome, even if BM is incubated 7 days at RT.

### 3.4. BM cfRNA Contains Stable miRNAs with a Similar Abundance Pattern as miRNA in BM Cells

**Figure 4:**
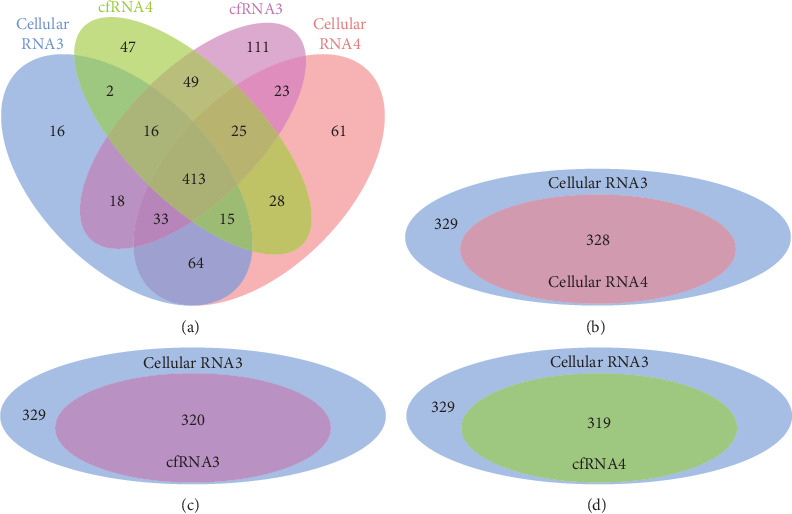
The BM supernatant contains stable miRNAs with as good integrity as in BM cells. (a) Cellular RNA (from cells in fresh BM of donors 3 and 4) and cfRNA (from fresh BM of donor 4 and RT-7-days BM of donor 3) were used to prepare the miRNA NGS library and sequence. After the bioinformatics analysis, detectable known human miRNAs based on miRBase 22.0 in different samples were compared. The graph shows the number of identical and different miRNAs between samples. To avoid the experimental bias in lower sequencing read data, detected miRNAs in cellular RNA3 whose normalized reads were greater than 50 were identified as a base pool; then, miRNAs in the base pool were compared with detectable miRNAs in other samples. The graphs show the numbers of identical miRNAs in the base pool and detectable miRNA in cellular (b) RNA4, (c) cfRNA3, and (d) cfRNA4.

It is reported that miRNAs are present in human plasma in a remarkably stable form [7]. Our results show that, like cfRNA in plasma [19], BM cfRNA is largely of low molecular weight (Supplemental Figure S2). To determine the existence and stability of cell-free miRNAs in human BM, cellular RNA (from fresh BM of donors 3 and 4) and cfRNA (from fresh BM of donor 4 and RT-7-days BM of donor 3) were used to generate libraries for NGS (next-generation sequencing) of miRNAs. The results of bioinformatics analysis show that 413 identical known human miRNAs can be detected in all samples (Figure 4(a)), and the relative trends of abundance of different miRNAs are considerably consistent between samples (Supplemental Table S2). We noticed that the specific miRNAs in different samples are all with very less read counts (Supplemental Table S3), and the missed detection of low-abundance miRNAs is often caused by the bias of the PCR process in library generation. To accurately compare abundance patterns of miRNA between samples, the number of detected miRNAs in cellular RNA3 whose normalized reads were greater than 50 was identified; then, these miRNAs were compared with detectable miRNAs from other samples. Results show there are 329 miRNAs with more than 50 normalized reads detected in cellular RNA3, which cover 320, 311, and 328 identical miRNAs detected in cfRNA3, cfRNA4, and cellular RNA4, respectively (Figures 4(b)–4(d)). Pearson analysis also suggests a strong correlation (*R* > 0.65) of detectable miRNAs between BM cellular RNA and cell-free RNA (Supplemental Figure S3b), notably between cfRNA3 and cfRNA4 or between cellular RNA 3 and 4 (*R* > 0.9). These data suggest that BM supernatant contains stable miRNAs whose abundance pattern is in accordance with miRNAs in BM cells and whose integrity can maintain pretty good even after BM is incubated at RT for 7 days. Moreover, abundance profiles of miRNAs in both cellular and cell-free RNAs show high similarity between different individuals.

### 3.5. Potential Candidate miRNAs in BM as Biomarkers of Early Breast Cancer

Changes of miRNA levels have been reported to be implicated in the initiation and progression of breast cancer [20, 21]. However, early traces of these changes are usually tiny and hard to be found. Certain basal levels could make it easier to detect anomalies. Our results of miRNA sequencing show that abundance patterns of miRNAs exhibit high similarity between BM samples from different mothers, no matter in BM cellular RNA or cfRNA (Figure S3b), which suggests a potential basal level might exist in healthy women. By comparing reported data about dysregulated miRNAs in breast cancer [22–25] and our miRNA sequencing data, we found that majority of reported miRNAs upregulated in breast cancer had a lower basal level and that majority of reported miRNAs downregulated in breast cancer had a higher basal level in BM cellular or cell-free RNA (Supplemental Table S4), despite opposite results in a few miRNAs. According to most reports and our miRNA basal abundance data, we got the inferential expression changes of dysregulated miRNAs in the initiation of breast cancer. Among these miRNAs, those with extremely low or high basal abundance in BM (listed in Table 1) could be chosen as potential candidates of biomarkers for research of early breast cancer screening, especially low-basal level miRNAs, because change from scratch is easier to detect.

## 4. Discussion

Our study reports the abundant existence and outstanding stability of BM cfNA (cfDNA and cell-free miRNA) which mainly originated from breast-derived cells and contains genetic information similar to that in BM cells. These characteristics make BM cfNA a potential noninvasive sample for the study of breast cancer risk and early detection. Early screening for breast cancer has always been paid much attention. Over the past years, tests of ctNA in plasma have gained wide attention for cancer diagnosis and early detection [26–30]. ctNA is released into circulation by apoptosis and necrosis of cells that occur more frequently as the tumor increases in volume [30, 31]. Thus, detectable ctNA existing in plasma often suggests that the tumor has developed considerably. In the initial stage of cancer, the fraction of ctNA in total cfNA is usually so small that it is hard to be detected.

Compared with that in plasma, cfNA in breast milk naturally has much more superiority in specificity and sensitivity of early breast cancer detection, because ctNA in blood would be extremely diluted by normal cfNA in circulation and constantly cleaned from the blood by the liver and kidney [30]. So there are far more chances for ctNA released from early breast tumor cells to be detected in BM than in plasma.

Our data shows that patterns of miRNA expression and DNA methylation exhibit high similarity between BM samples from different mothers, which suggests a potential basal level exists in healthy women. Changes of miRNA levels and DNA methylation have been reported to be implicated in the initiation and progression of breast cancer [20, 21, 32–34]. However, early traces of these changes are usually unconspicuous and hard to be found. Certain basal levels could make it easier to detect anomalies. Development of breast cancer is a slow and gradual process that begins with sporadic neoplastic cells [35, 36]. It takes several decades for dysplasia cells to become a detectable tumor mass with metastatic potential [36–39]. In such a long initial period, early abnormalities of miRNA or DNA methylation are difficult to be found by monitoring plasma cfNA due to the challenge in specificity and sensitivity but are likely to be found in BM cfNA that mainly comes from breast-derived cells. In Asia, especially in China, the incidence of breast cancer increased rapidly with age after 20 years, and the first age peak is 45–55 years [2–4]. Thus, women aged 25-40 years are a high-risk group that could suffer from the asymptomatic period of breast cancer. And this age span is precisely the fertility and lactation peak of women, so breastfeeding mothers could benefit from detection of early alteration of BM cfDNA or cell-free miRNA. Noninvasive and convenient sampling make it possible to obtain a large sample for studying and establishing the thresholds of miRNA and DNA methylation basal levels in healthy women, which would be greatly helpful for studying variations associated with the initiation and progression of breast cancer.

Our study also raises questions about mechanisms of BM cfDNA and miRNA stability and cfNA releasing into BM from cells in vitro. The remarkable stability of cfDNA and miRNA in BM is very advantageous for long-distance transportation of BM samples. Research of the mechanism by which cfNAs keep intactness in BM could help us deeply understand this heterogeneous liquid and find enlightenment for the protection of cfNA samples. One possibility is that exosomes exist in BM and package cfNA inside like in plasma [40].

Our results indicate that cfDNA with a major size of ~180 bp can be generated in vitro after incubation of BM at RT (Figure 1(c)). The most possible explanation of this intriguing phenomenon is the release of fragmented DNA from apoptotic cells in BM. If that is the case, what components in BM induce the apoptosis? The efficient clearance of cells in BM also raises provocative questions regarding the potential existence of specifically induced programmed cell degradation in BM. Since most of the cells in milk are dead or dying cells, whether there is a mechanism to induce apoptotic degradation of dead cells, additional studies will be needed to explore the answers of these questions.

## 5. Conclusions

Ideal sample sources for detecting early nucleic acid changes are greatly desired to reduce the morbidity and mortality caused by breast cancer. We show here that cell-free DNA and microRNA exist in BM in a relatively stable form at RT; these cfNAs in BM mainly come from breast-derived cells and contain genetic information similar to that in BM cells. Perhaps, these characteristics might make BM cfNAs new noninvasive sample sources for searching early alterations of nucleic acid associated with the initiation and progression of breast cancer.

## Figures and Tables

**Table 1:** Dysregulated miRNAs in breast cancer with extremely low or high basal abundance in BM. The inferential expression changes in early breast cancer (BC) are analyzed according to reports and basal abundance data from miRNA sequencing.

miRNA name	miRNA sequence	Normalized reads				Inferential expression in early BC
		cfRNA4	cfRNA3	cellRNA3	cellRNA4	
hsa-miR-25-5p_R+2	AGGCGGAGACTTGGGCAATTGCT	35	42	28	43	Upregulated
hsa-miR-34a-3p_R+1	CAATCAGCAAGTATACTGCCCTT	0	0	0	8	Upregulated
hsa-miR-92a-2-5p	GGGTGGGGATTTGTTGCATTAC	7	0	0	0	Upregulated
hsa-miR-103a-2-5p	AGCTTCTTTACAGTGCTGCCTTG	36	7	53	43	Upregulated
hsa-miR-125b-1-3p	ACGGGTTAGGCTCTTGGGAGCT	10	25	10	10	Upregulated
hsa-miR-133a-3p_L-1R+1	TTGGTCCCCTTCAACCAGCTGT	8	4	7	2	Upregulated
hsa-miR-155-3p	CTCCTACATATTAGCATTAACA	0	0	5	4	Upregulated
hsa-miR-191-3p_R-3	GCTGCGCTTGGATTTCGTC	30	14	7	0	Upregulated
hsa-miR-192-3p_R+1	CTGCCAATTCCATAGGTCACAGT	0	0	0	4	Upregulated
hsa-miR-195-3p_R-2	CCAATATTGGCTGTGCTGCT	50	42	43	40	Upregulated
hsa-miR-199a-5p_R-1	CCCAGTGTTCAGACTACCTGTT	22	23	20	19	Upregulated
hsa-miR-200c-5p	CGTCTTACCCAGCAGTGTTTGG	18	6	65	25	Upregulated
hsa-miR-202	AGAGGTATAGCGCATGGGAA	0	0	0	0	Upregulated
hsa-miR-215-5p_R+1	ATGACCTATGAATTGACAGACA	0	0	18	26	Upregulated
hsa-mir-222-p5	AAGGTGTAGGTACCCTCAAT	0	0	8	16	Upregulated
hsa-miR-299-5p	TGGTTTACCGTCCCACATACAT	0	0	0	0	Upregulated
hsa-miR-373	GAAGTGCTTCGATTTTGGGGTGT	0	0	0	0	Upregulated
hsa-miR-375-5p	GCGACGAGCCCCTCGCACAAACC	19	12	0	0	Upregulated
hsa-miR-376c	AACATAGAGGAAATTCCACGT	0	0	0	0	Upregulated
hsa-miR-382-5p	GAAGTTGTTCGTGGTGGATTCG	17	0	0	10	Upregulated
hsa-miR-382-3p_R+1	AATCATTCACGGACAACACTTT	0	0	0	4	Upregulated
hsa-miR-409-5p_R-1	AGGTTACCCGAGCAACTTTGCA	14	0	0	23	Upregulated
hsa-miR-409-3p_L+1	CGAATGTTGCTCGGTGAACCCCT	0	0	0	6	Upregulated
hsa-miR-523-3p_L-1R-2	AACGCGCTTCCCTATAGAGG	11	19	8	0	Upregulated
hsa-miR-526b-5p_R-1	CTCTTGAGGGAAGCACTTTCTG	7	25	32	14	Upregulated
hsa-miR-625-5p_R-1	AGGGGGAAAGTTCTATAGTC	7	0	15	12	Upregulated
hsa-let-7a-5p	TGAGGTAGTAGGTTGTATAGTT	69874	57909	83431	129086	Downregulated
hsa-let-7b-5p	TGAGGTAGTAGGTTGTGTGGTT	82701	116853	81208	48587	Downregulated
hsa-miR-30a-5p	TGTAAACATCCTCGACTGGAAG	143946	97103	178925	183651	Downregulated
hsa-miR-30b-5p	TGTAAACATCCTACACTCAGCT	383303	264659	372314	346935	Downregulated
hsa-miR-141-3p_R-1	TAACACTGTCTGGTAAAGATG	45058	66296	119600	65598	Downregulated
hsa-miR-148a-3p	TCAGTGCACTACAGAACTTTGT	386987	588485	918411	876058	Downregulated
hsa-miR-148b-3p_R-1	TCAGTGCATCACAGAACTTTG	20149	21809	40891	61755	Downregulated
hsa-miR-200c-3p	TAATACTGCCGGGTAATGATGGA	764438	473643	254207	253470	Downregulated
hsa-miR-200b-3p	TAATACTGCCTGGTAATGATGA	354432	251845	266377	258231	Downregulated
hsa-miR-200a-3p_R+1	TAACACTGTCTGGTAACGATGTT	345502	350091	385973	393857	Downregulated
hsa-miR-335-5p_R-2	TCAAGAGCAATAACGAAAAAT	27462	21489	35648	31513	Downregulated
hsa-miR-375-3p	TTTGTTCGTTCGGCTCGCGTGA	170762	305772	53533	42830	Downregulated

## Data Availability

The data used to support the findings of this study are included within the supplementary information file.

## Supplementary Materials

Supplementary Materials Supplementary text of materials and methods, including full details of the experiments and result processing. Supplementary figures: Figure S1: genomic DNA (gDNA) of BM cells was easily degraded when BM was stored at room temperature. 10 mL of whole BM samples (from donors 3 and 4) was incubated at room temperature for the indicated time; then, BM cells were isolated and gDNA was extracted detected by agarose gel electrophoresis. Figure S2: identification of the existence and stability of cfRNA in human BM. (a) cfRNA extracted from fresh BM samples. (b) cfRNA extracted from the RT-2-days BM sample. (c) cfRNA extracted from the RT-7-days BM sample. (d) Total RNA isolated from fresh BM cells. RNA and cfRNA were detected by Agilent 2100 Bioanalyzer. The BM samples used in this experiment are from donor 3. Figure S3: pearson correlation between samples. (**a**) Pearson correlation of methylation between indicated samples according to data of whole genome bisulfite sequencing (WGBS). (**b**) Pearson correlation of miRNA abundance profiles between indicated samples according to the normalized reads of 413 identical known human miRNAs detectable in all samples. Supplementary tables: Table S1: forward and reverse primers used in real-time PCR. Table S2: partial data of normalized reads of detectable miRNAs in all samples. Table S3: partial data of normalized reads of miRNAs which are only detectable in partial samples. Table S4: previous reported microRNAs dysregulated in breast cancer were listed and compared with our miRNA basal abundance data. The inferential expression changes of miRNAs in early breast cancer (BC) are analyzed according to reports and our miRNA sequencing data.
