# Supplementary material for: Potential of Using Cell-Free DNA and miRNA in Breast Milk to Screen Early Breast Cancer
Source: Biomed Res Int. 2020 Jul 2;2020:8126176. doi: 10.1155/2020/8126176 (PMC7354639; doi:10.1155/2020/8126176)
Supplement: Supplementary Materials — Supplementary text of materials and methods, including full details of the experiments and result processing. Supplementary figures: Figure S1: genomic DNA (gDNA) of BM cells was easily degraded when BM was stored at room temperature. 10 mL of whole BM samples (from donors 3 and 4) was incubated at room temperature for the indicated time; then, BM cells were isolated and gDNA was extracted detected by agarose gel electrophoresis. Figure S2: identification of the existence and stability of cfRNA in human BM. (a) cfRNA extracted from fresh BM samples. (b) cfRNA extracted from the RT-2-days BM sample. (c) cfRNA extracted from the RT-7-days BM sample. (d) Total RNA isolated from fresh BM cells. RNA and cfRNA were detected by Agilent 2100 Bioanalyzer. The BM samples used in this experiment are from donor 3. Figure S3: pearson correlation between samples. (a) Pearson correlation of methylation between indicated samples according to data of whole genome bisulfite sequencing (WGBS). (b) Pearson correlation of miRNA abundance profiles between indicated samples according to the normalized reads of 413 identical known human miRNAs detectable in all samples. Supplementary tables: Table S1: forward and reverse primers used in real-time PCR. Table S2: partial data of normalized reads of detectable miRNAs in all samples. Table S3: partial data of normalized reads of miRNAs which are only detectable in partial samples. Table S4: previous reported microRNAs dysregulated in breast cancer were listed and compared with our miRNA basal abundance data. The inferential expression changes of miRNAs in early breast cancer (BC) are analyzed according to reports and our miRNA sequencing data. [file 8126176.f1.pdf]

Supplemental material for

Potential of Using cell-free DNA and miRNA in breast milk to Study Breast Cancer Risk

Qinghe Song, Yingying Zhang, Hongtao Liu, Yuguang Du

Supplemental Text

Human donor breast milk samples collection. Each donor was asked to provide at least 100 mL of breast milk (BM) by pumping or hand expressing. Breast milk was processed for BM cells isolation within 2 h of collection using the protocol described behind. Whole BM for stability experiments were packed into 15 ml centrifuge tubes (JetBiofil) and stored at room temperature.

Processing of milk samples and Isolation of BM cells and BM supernatant. To separate the BM cells, fresh breast milk samples were centrifuged immediately on arrival at the laboratory at 1,500 G for 10 min in 15 mL plastic centrifuge tubes. After removal the milk fat by aspiration, the supernatant was transferred to new tubes, the cell pellet was washed in ~5 mL PBS one time, then used for isolation of RNA via traditional TRIZol (Ambion) method, and extraction of genomic DNA (gDNA) via the QIAamp DNA mini kit (Qiagen). The newly transferred supernatant was centrifuged again at 10,000g at RT for 10 min, removed milk fat and collected BM supernatant for stability experiments and future extraction of cell-free nucleic acid. BM cfDNA was extracted using a cfDNA purification Kit (SummerBio). BM cfRNA isolation was carried out using a cfRNA purification Kit (SummerBio). All nucleic acid extraction processes are carried out following the manufacturer's procedure.

Stability experiments of BM cells, cell-free DNA and miRNA. For BM cells stability test, whole BM from donor 3 and 4 was packed by 10ml each into centrifuge tubes. After incubation for designed time at RT, BM cells were separated to isolate gDNA for detection. For cfDNA stability test, whole BM and BM supernatant from donor 1, 2, 3, 4 and 5 were packed by 2ml each into centrifuge tubes and incubation for designed time at RT, then isolated BM supernatant was used to extract cfDNA for detection of abundance by qRT-PCR. For cell-free miRNA stability test, 2ml of whole BM from donor 3 was incubated 7 days at RT, then isolated BM supernatant to extract cfRNA for generating NGS libraries of miRNA. All samples for incubation at RT were added with sodium azide at 0.05% final concentration as antimicrobial.

Nucleic acid Extraction Procedures. Isolation of cellular RNA via traditional TRIZol (Ambion) method, extraction of genomic DNA (gDNA) via the QIAamp DNA mini kit (Qiagen). BM cfDNA was extracted using a cfDNA purification Kit (SummerBio). BM cfRNA isolation was carried out using a cfRNA purification Kit (SummerBio). All nucleic acid extraction processes are carried out following the manufacturer's procedure.

Measurement of cfDNA abundance in BM by Using qRT-PCR Assays. To accurately measure abundance of cfDNA, quantitative real-time PCR method is designed. We choose human long interspersed nuclear elements (LINE) as target amplification region and designed forward primer (5' CAAGTCCTGAGTGACCTAC 3') and reverse primer (5' GTCTCGTTGATCTGTCTAATG 3'). Human DNA Standard (Thermo) was used to generate standard curve. cfDNA samples and diluted DNA standards were subjected to qRT-PCR as

follows: initial denaturation at 95 ℃ for 10 min, then (95 ℃ for 10 s, 60 ℃ for 20 s) x 40 cycles, and default melt curve setting. Ct values of gradient standards were used to generate standard curve, to which concentrations of cfDNA samples were calculated according. Then concentration of cfDNA was normalized into ng cfDNA per milliliter of BM by converting sample volume of BM for extracting cfDNA and final elution volume

Measurement of RNA difference between BM cellullar RNA and BM cfRNA By Using RT-qPCR Assays. RNA samples from different donors were subjected to RT-qPCR Assays to determine the difference of expression levels of LALBA, K-CSN, B-CSN, ELF5, TACSTD2 and PPBP, CD99, ITGB3, RGS18, CD45. Reverse transcription was performed using SumScript 1st strand cDNA synthesis kit (SummerBio). qPCR analysis was carried out at a CFX connect Real-time PCR instrument (BioRad Laboratories) using SYBR QPCR Mixture (SummerBio). Forward and reverse primers listed in Table S1. Thermal cycling was set up as the following condition: 95 ℃ for 10 min, followed by 40 cycles of 10 s at 95 ℃, 20 s at 60 ℃, and default melt curve setting. The comparative Ct method (-ΔCt) was used to determine the relative expression levels of each gene. -ΔCt was calculated by formula of $-[Ct_{(Target\ gene)}-Ct_{(GAPDH)}]$.

Whole Genome Bisulfite Sequencing (WGBS) Library Generation and NGS data analysis were performed by LC Sciences (Houston, TX, USA). The brief process is as follows.

WGBS Library construction and sequencing. cfDNA and sonication-fragmented DNA samples were subjected to bisulfite conversion. The Accel-NGS Methyl-Seq DNA Library Kit (Swift, MI, USA) was utilized for attaching adapters to single-stranded DNA fragments. Briefly, the Adaptase step is a highly efficient, proprietary reaction that simultaneously performs end repair, tailing of 3’ ends, and ligation of the first truncated adapter complement to 3’ ends. The Extension step is used to incorporate truncated adapter 1 by a primer extension reaction. The Ligation step is used to add the second truncated adapter to the bottom strand only. The Indexing PCR step increases yield and incorporates full length adapters. Bead-based SPRI clean-ups are used to remove both oligonucleotides and small fragments, as well as to change enzymatic buffer composition. Finally, the pair-end 2×150bp sequencing was performed on an illumina Hiseq 4000 platform.

WGBS Bioinformatics analysis. Firstly, Cutadapt [1] and perl scripts in house were used to remove the reads that contained adapter contamination, low quality bases and undetermined bases. Then sequence quality was verified using FastQC (http://www.bioinformatics. babraham.ac.uk/projects/fastqc/). Reads that passed quality control were mapped to reference genome using WALT [2]. After alignment, reads were further deduplicated using samtool [3]. For each cytosine site (or guanine corresponding to a cytosine on the opposite strand) in the reference genome sequence, the DNA methylation level was determined by the ratio of the number of reads supporting C (methylated) to that of total reads (methylated and unmethylated) using per scripts in house and MethPipe [4]. Differentially methylated regions (DMRs) were calculated by R package-MethylKit [5] with default parameters (1000 bp slide windows, 500 bp overlap, p value < 0.05).

Whole Genome Sequencing(WGS) Library Generation and NGS data analysis for cfDNA. cfDNA samples were directly used for WGS library generation by utilizing NEBNext Ultra DNA Library Prep Kit for Illumina (NEB #E7370) according to manufacturer’s

recommended protocol. After passing the quality control, libraries were subjected to the pair-end 2×150bp sequencing on an illumina Hiseq 4000 platform. Alignment analysis for raw reads were processed by the Burrows-Wheeler Aligner software to simply determine the chromosomal origin of each sequenced DNA fragments. we counted only sequences that could be mapped to just one location in the hg19 reference human genome with no mismatch [6]. To reduce GC bias introduced by the PCR process, we applied weight to each sequenced read based on the local genomic GC content of large regions of the human genome with relatively homogeneous GC content [7]. We first got the read count for bins of size of 500kb, then averaged the counts in each 0.5% of GC content bin. Counts in each GC content bin is weighted by $W_i = \bar{M} / M_i$, where $\bar{M}$ is the average counts across all GC content bins, and Mi is the average count in bin i. Counts ratio is normalized by *500kb bin counts/ all counts*, and a reference baseline of mean of counts ratios based on a set of normal diploid cfDNAs was created. Copy number of cfDNA samples was calculated by *sample counts ratios/ reference mean of counts ratios*, which was further plotted into genome-wide copy number for data visualization using ggplot2 [8]. Z-score was calculated based on the same reference data set by referring to previous reports [9].

miRNA Sequencing and Bioinformatics analysis. Service of miRNA library generation and NGS data analysis was provided by LC Sciences (Houston, Texas, USA). In brief, miRNA library was prepared by TruSeq Small RNA Sample Prep Kits (Illumina) and single-end sequencing (36 bp or 50 bp) was performed on an Illumina Hiseq 2500 following the vendor's recommended protocol. Raw reads were subjected to the Illumina pipeline flter (Solexa 0.3), and then the dataset was further processed with an in-house program, ACGT101-miR (LC Sciences) to remove adapter dimers, junk, low complexity, common RNA families (rRNA, tRNA, snRNA, snoRNA) and repeats. Subsequently, unique sequences with length in 18~26 nucleotide were mapped to human species precursors in miRBase 22.0 by BLAST search to identify known miRNAs and novel 3p- and 5p- derived miRNAs. Length variation at both 3. and 5. ends and one mismatch inside of the sequence were allowed in the alignment. The unique sequences mapping to human species mature miRNAs in hairpin arms were identified as known miRNAs. The unique sequences mapping to the other arm of known human species precursor hairpin opposite to the annotated mature miRNA-containing arm were considered to be novel 5p- or 3p- derived miRNA candidates. The remaining sequences were mapped to other selected species precursors (with the exclusion of human species) in miRBase 22.0 by BLAST search, and the mapped pre-miRNAs were further BLASTed against the human species genomes to determine their genomic locations. The above two we defined as known miRNAs.

Supplementary Figures

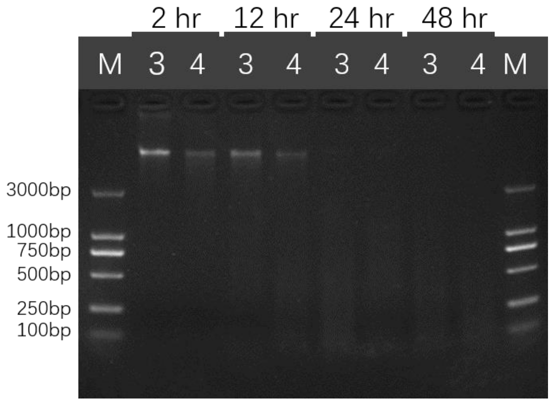

Figure S1: genomic DNA (gDNA) of BM cells was easily degraded when BM was stored at room temperature. 10mL of Whole BM samples (from donor 3 and 4) were incubated at room temperature for indicated time, then BM cells was isolated and gDNA was extracted detected by agarose gel electrophoresis.

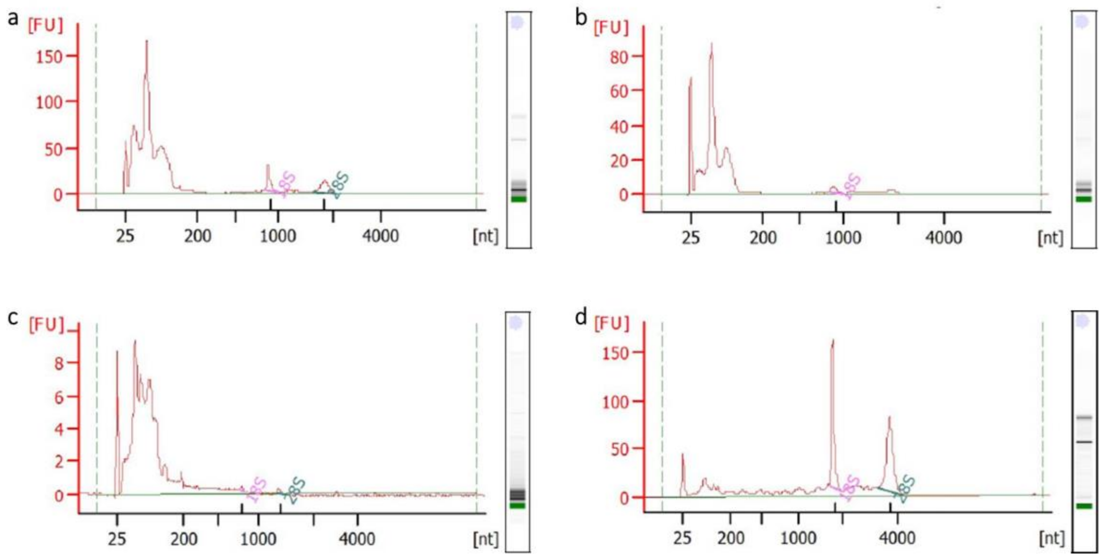

Figure S2: Identification of existence and stability of cfRNA in human BM. (a) cfRNA extracted from fresh BM samples; (b) cfRNA extracted from RT-2-days BM sample; (c) cfRNA extracted from RT-7-days BM sample; (d) Total RNA isolated from fresh BM cells. RNA and cfRNA were detected by Agilent 2100 Bioanalyzer. The BM samples used in this experiment are from donor 3.

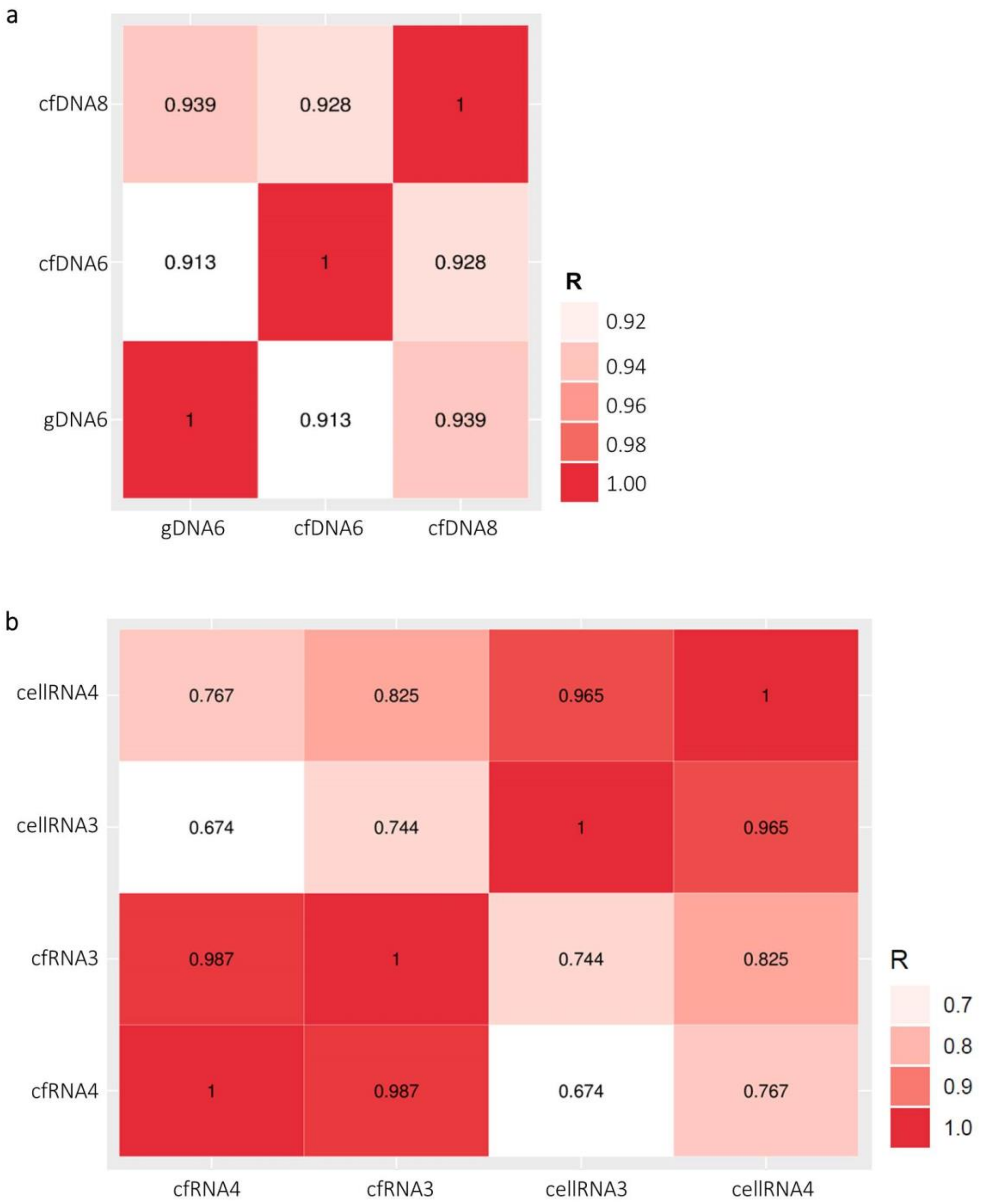

Figure S3: Pearson correlation between samples. (A) Pearson correlation of methylation between indicated samples according to data of Whole Genome Bisulfite Sequencing (WGBS). (B) Pearson correlation of miRNA abundance profiles between indicated samples according to the normalized reads of 413 identical known human miRNAs detectable in all samples.

Supplementary Tables

Table S1: Forward and reverse primers used in real-time PCR.

Genes	Forward primers	Reverse primers

LALBA	CCTGGATGATGACATTACTGA	ACACTCACAACTTCTCACAA
B-CSN	GAGGATGAACACCAGGATAA	TGAGCAAGAGGCAGAATG
K-CSN	ATTCCTCAGCGGCAATAC	GGTTCAACAGTAGCAATGG
ELF5	ACTTCCACAGACATCCTTC	ATGCCAGCCATCAGTTAG
TACSTD2	CACGCTTCCTGATTCCTC	AATGCTTCCTTCAAGACAGA
PPBP	TAGACAGTGACTTGTATGCT	ATCCTTCAGTGTGGCTATC
RGS18	GACACATTCATCTCTACAGAAC	CGGTGATAAGGTAGTCTACTT
ITGB3	TTAGCAGCCAGTCTTGAAG	AGAGAATCTTAACCGCACAT
CD99	ATAGAAGATTGTCGGCAGAA	CAAAGCACATCGCAAGATT
CD45	TGCTCAGAATGGACAAGTAA	ACCTTCAGCCTGTTCCTT
GAPDH	TCTCTGCTCCTCCTGTTC	GACTCCGACCTTCACCTT

LALBA: lactalbumin, alpha; B-CSN: casein beta; K-CSN: casein kappa; ELF5: E74-like factor 5; TACSTD2: tumor-associated calcium signal transducer 2; SPP1: secreted phosphoprotein 1; ELF5: E74-like factor 5; PPBP: pro-platelet basic protein; RGS18: regulator of G-protein signaling 18; ITGB3: integrin, beta 3; CD99: Cluster of differentiation 99; CD45: Cluster of differentiation 45; GAPDH: glyceraldehyde-3-phosphate dehydrogenase.

Table S2: Partial data of normalized reads of detectable miRNAs in all samples.

miRNA name	miRNA sequence	Normalized reads			
		cfRNA4	cfRNA3	cellRNA3	cellRNA4
hsa-miR-1-3p	TGGAATGTAAAGAAGTATGTAT	13.84	40.95	32.43	15.32
hsa-let-7f-5p	TGAGGTAGTAGATTGTATAGTT	73829.24	39642.02	169680.29	251397.85
hsa-let-7f-1-3p_R-1_1ss21CT	CTATACAATCTATTGCCTTCT	142.51	127.52	245.32	132.35
hsa-let-7d-5p	AGAGGTAGTAGGTTGCATAGTT	23649.91	16202.40	15598.37	19948.15
hsa-let-7d-3p_R-2	CTATACGACCTGCTGCCTTT	10422.37	12390.31	4978.73	3429.99
hsa-let-7e-5p	TGAGGTAGGAGGTTGTATAGTT	11059.61	3558.48	3586.36	10139.19
hsa-let-7e-3p_R-2	CTATACGGCCTCCTAGCTTT	89.93	108.80	91.17	89.16
hsa-let-7a-5p	TGAGGTAGTAGGTTGTATAGTT	69874.08	57908.58	83430.84	129086.21
hsa-let-7a-3p_R-1	CTATACAATCTACTGTCTTT	253.42	427.21	932.40	470.43
hsa-miR-7-5p	TGGAAGACTAGTGATTTTGTTGTT	303.00	168.85	732.19	2653.07
hsa-let-7i-5p	TGAGGTAGTAGTTTGTGCTGTT	10651.35	8548.39	36036.83	72137.46
hsa-let-7i-3p	CTGCGCAAGCTACTGCCTTGCT	318.22	445.73	1184.29	759.98
hsa-let-7c-5p	TGAGGTAGTAGGTTGTATGGTT	2273.01	2554.75	2570.07	2421.30
hsa-let-7g-5p	TGAGGTAGTAGTTTGTACAGTT	95427.35	76654.95	254574.04	418970.55
hsa-let-7g-3p_R-1	CTGTACAGGCCACTGCCTTG	193.70	63.17	276.34	149.07
hsa-let-7b-5p	TGAGGTAGTAGGTTGTGTGGTT	82701.24	116853.47	81207.83	48586.97
hsa-let-7b-3p_R-1_1ss21CT	CTATACAACCTACTGCCTTCT	3748.07	2484.85	1170.19	601.85
hsa-miR-7-1-3p	CAACAAATCACAGTCTGCCATA	135.59	166.12	166.37	216.64
hsa-let-7f-2-3p_1ss22CT	CTATACAGTCTACTGTCTTTCT	5.07	1.95	142.87	85.91
hsa-miR-9-5p	TCTTTGGTTATCTAGCTGTATGA	271.64	224.62	318.00	137.23
hsa-miR-10a-5p_R-1	TACCCTGTAGATCCGAATTTGT	18065.16	11759.74	4595.25	8784.65
hsa-miR-10b-5p_R-1	TACCCTGTAGAACCGAATTTGT	182.63	122.84	69.55	87.77
hsa-miR-15a-5p	TAGCAGCACATAATGGTTTGTG	135.59	272.58	1843.17	1779.78
hsa-miR-15b-5p	TAGCAGCACATCATGGTTTACA	1285.33	827.11	2492.66	2148.97

hsa-miR-15b-3p_R-1	CGAATCATTATTTGCTGCTCT	59.49	63.17	216.18	376.85
hsa-miR-16-5p	TAGCAGCACGTAAATATTGGCG	7283.76	7890.33	12244.75	13131.01
hsa-miR-16-2-3p_L+1R-1	ACCAATATTACTGTGCTGCTTT	221.37	391.91	563.95	693.80
hsa-miR-17-5p	CAAAGTGCTTACAGTGCAGGTAG	1691.40	1989.98	7665.48	6633.94
hsa-miR-17-3p	ACTGCAGTGAAGGCACTTGTAG	134.21	116.99	531.05	475.07
hsa-miR-18a-5p	TAAGGTGCATCTAGTGCAGATAG	81.63	91.25	794.23	714.00
hsa-miR-19b-3p_R-1	TGTGCAAATCCATGCAAAACTG	549.97	624.14	3133.21	3018.31
hsa-miR-19a-3p	TGTGCAAATCTATGCAAAACTGA	196.47	291.30	1393.89	1409.19
hsa-miR-20a-5p	TAAAGTGCTTATAGTGCAGGTAG	4964.22	3870.00	31801.10	31943.72
hsa-miR-20a-3p	ACTGCATTATGAGCACTTAAAG	16.60	24.57	155.09	113.54
hsa-miR-21-5p	TAGCTTATCAGACTGATGTTGA	167597.73	225676.62	1216907.49	773411.75
hsa-miR-21-3p_R+1	CAACACCAGTCGATGGGCTGTC	123.14	188.35	2935.36	2381.63
hsa-miR-22-5p_R-1	AGTTCTTCAGTGGCAAGCTTT	3782.66	5124.12	3181.61	2292.47
hsa-miR-22-3p	AAGCTGCCAGTTGAAGAACTGT	161231.96	171429.96	128999.63	88835.69
hsa-miR-23a-5p_R-1	GGGGTTCCTGGGGATGGGATT	19.37	104.12	9.40	9.75
hsa-miR-23a-3p_R-1	ATCACATTGCCAGGGATTTC	3745.99	4037.88	32975.05	25369.68
hsa-miR-23b-3p_R-5	ATCACATTGCCAGGGATT	729.83	697.84	3237.07	1712.21
hsa-miR-24-3p_R-2	TGGCTCAGTTCAGCAGGAAC	3255.52	4550.88	15252.95	7701.11
hsa-miR-24-2-5p_L+1R-1	GTGCCTACTGAGCTGAAACACA	224.14	331.08	775.90	807.34
hsa-miR-25-5p_R+2	AGGCGGAGACTTGGGCAATTGCT	34.59	42.12	28.20	43.19
hsa-miR-25-3p	CATTGCACTTGTCTCGGTCTGA	24937.31	34049.67	24623.90	23471.49
hsa-miR-26b-5p_R+1	TTCAAGTAATTCAGGATAGGTT	37110.81	43039.89	98969.64	81495.06
hsa-miR-26b-3p	CCTGTTCTCCATTACTTGGCT	114.84	70.19	112.79	137.23
hsa-miR-26a-5p	TTCAAGTAATCCAGGATAGGCT	60712.99	45029.88	117750.56	109287.12
hsa-miR-26a-2-3p_R-1	CCTATTCTTGATTACTTGTTT	34.59	24.57	179.52	94.04
hsa-miR-27a-5p	AGGGCTTAGCTGCTTGTGAGCA	85.78	104.12	578.05	822.67
hsa-miR-27a-3p_R-1	TTCACAGTGGCTAAGTTCCG	4813.64	12190.85	79125.52	29570.33
hsa-miR-27b-3p_R-1	TTCACAGTGGCTAAGTTCTG	2902.94	3878.77	17108.50	7536.25
hsa-miR-28-5p_R-1	AAGGAGCTCACAGTCTATTGA	2992.18	2159.03	5951.70	4937.64
hsa-miR-28-3p	CACTAGATTGTGAGCTCCTGGA	6856.93	4332.11	1423.03	5482.83
hsa-miR-29a-3p_R-1	TAGCACCATCTGAAATCGGTT	65680.20	92681.09	207835.42	98081.72
hsa-miR-29b-3p_R-3	TAGCACCATTTGAAATCAGT	2256.36	3032.36	5154.10	2840.22
hsa-miR-29c-5p_R-1	TGACCGATTTCTCCTGGTGTT	672.41	541.66	1325.28	1090.85
hsa-miR-29c-3p_R-1	TAGCACCATTTGAAATCGGTT	2998.87	5932.52	15753.37	7486.56
hsa-miR-29b-2-5p_R+1	CTGGTTTCACATGGTGGCTTAGA	358.34	604.83	568.65	1151.46
hsa-miR-30b-5p	TGTAAACATCCTACACTCAGCT	383302.82	264658.95	372313.79	346934.96
hsa-miR-30b-3p_R-2	CTGGGAGGTGGATGTTTACT	478.71	377.87	203.96	172.06
hsa-miR-30c-5p_R+1	TGTAAACATCCTACACTCTCAGCT	54995.42	46195.28	42790.74	47149.16
hsa-miR-30c-2-3p_R-2	CTGGGAGAAGGCTGTTTACT	80.25	46.80	146.63	242.41
hsa-miR-30a-5p	TGTAAACATCCTCGACTGGAAG	143946.44	97103.28	178924.64	183650.88
hsa-miR-30a-3p_1ss22CT	CTTTCAGTCGGATGTTTGCAGT	3262.44	3162.80	3578.73	5698.78
hsa-miR-30d-5p	TGTAAACATCCCCGACTGGAAG	3694759.6	2510709.18	1131940.55	1230804.8
hsa-miR-30d-3p_R-2	CTTTCAGTCAGATGTTTGCT	1120.68	1056.41	1885.94	1538.41
hsa-miR-30e-5p_R+2	TGTAAACATCCTTGACTGGAAGCT	63270.96	44668.19	92393.68	156105.41

hsa-miR-30e-3p_1ss22CT	CTTTCAGTCGGATGTTTACAGT	2883.34	4249.63	4055.73	9630.66
hsa-miR-32-5p_R-2	TATTGCACATTACTAAGTTG	408.15	1109.06	3934.49	1496.27
hsa-miR-32-3p_R-1	CAATTTAGTGTGTGTGATATT	42.89	64.34	143.81	128.17
hsa-miR-33a-3p_R-1	CAATGTTTCCACAGTGCATCA	15.22	26.91	121.25	107.27
hsa-miR-33b-3p_R-1	CAGTGCCTCGGCAGTGCAGCC	304.38	214.09	86.47	71.75
hsa-miR-34a-5p_R+1	TGGCAGTGTCTTAGCTGGTTGTT	95.47	113.48	1263.25	1480.94
hsa-miR-92b-3p_R-2	TATTGCACTCGTCCCGGCCT	3241.68	2805.40	802.06	544.03
hsa-miR-92a-1-5p	AGGTTGGGATCGGTTGCAATGCT	24.90	65.51	25.38	34.83
hsa-miR-92a-3p	TATTGCACTTGTCCCGGCCTGT	71133.05	52695.39	36107.63	24183.40
hsa-miR-93-5p	CAAAGTGCTGTTCGTGCAGGTAG	7586.07	6588.83	12813.87	14362.22
hsa-miR-93-3p_R+1	ACTGCTGAGCTAGCACTTCCCGA	166.03	277.26	158.85	137.92
hsa-miR-96-5p_R-2	TTTGGCACTAGCACATTTTTG	12768.89	12150.49	16593.27	26239.02
hsa-miR-98-5p	TGAGGTAGTAAGTTGTATTGTT	2425.25	1655.57	5080.95	7802.61
hsa-miR-98-3p_L-1R+1_1ss22CT	TATACAACTTACTACTTTCCTT	52.58	93.59	89.29	60.60
hsa-miR-99a-5p_R-1	AACCCGTAGATCCGATCTTGT	161476.85	114593.15	62097.86	45214.04
hsa-miR-99a-3p_R+1	CAAGCTCGCTTCTATGGGTCTGA	98.23	87.74	112.79	80.80
hsa-miR-99b-5p	CACCCGTAGAACCGACCTTGCG	220722.34	112993.91	19611.33	48173.84
hsa-miR-99b-3p_R-2	CAAGCTCGTGTCTGTGGGTC	864.73	765.11	423.90	803.86
hsa-miR-100-5p	AACCCGTAGATCCGAACTTGTG	11176.41	5719.60	2669.83	4109.16
hsa-miR-101-5p_L+1R-2	TCAGTTATCACAGTGCTGATG	8.30	8.19	55.46	20.20
hsa-miR-101-3p_R+1	TACAGTACTGTGATAACTGAAG	16836.56	29211.00	136941.91	79067.46
hsa-miR-103a-2-5p	AGCTTCTTTACAGTGCTGCCTTG	35.97	7.02	52.64	43.19
hsa-miR-103a-3p	AGCAGCATTGTACAGGGCTATGA	38637.80	22561.93	35255.44	76849.76
hsa-miR-106b-5p	TAAAGTGCTGACAGTGCAGAT	909.00	2044.97	8091.73	5302.42
hsa-miR-106b-3p_L+2R-2	TACCGCACTGTGGGTACTTGCT	2252.44	2146.75	1731.32	2544.63
hsa-miR-106a-5p_1ss1AC	CAAAGTGCTTACAGTGCAGGTAG	1691.40	1989.98	7665.48	6633.94
hsa-miR-107_R-2	AGCAGCATTGTACAGGGCTAT	2940.53	3355.25	6352.57	5061.63
hsa-miR-125b-5p_R-1	TCCCTGAGACCCTAACTTGTG	29145.42	29130.87	18317.07	8758.18
hsa-miR-125b-2-3p_L-2	ACAAGTCAGGCTCTTGGGAC	936.67	560.38	443.64	335.75
hsa-miR-125a-5p_R-1	TCCCTGAGACCCTTTAACCTGTG	142400.31	113799.96	35452.67	40911.92
hsa-miR-125a-3p_R-1	ACAGGTGAGGTTCTTGGGAGC	799.70	659.82	62.97	93.34
hsa-miR-125b-1-3p	ACGGGTTAGGCTCTTGGGAGCT	9.68	24.57	10.34	9.75

Table S3: Partial data of normalized reads of miRNAs that only detectable in partial samples.

miRNA name	miRNA sequence	Normalized reads			
		cfRNA4	cfRNA3	cellRNA3	cellRNA4
hsa-miR-15a-3p_1ss22AT	CAGGCCATATTGTGCTGCCTCT	0	0	4.70	0
hsa-miR-18b-5p_R-4	TAAGGTGCATCTAGTGCAG	0	0	3.76	0
hsa-miR-23b-5p_L-1R-3	GGGTTCCTGGCATGCTGA	0	23.40	0	0
hsa-miR-26a-1-3p_R+1	CCTATTCTTGGTTACTTGCACGA	0	9.36	4.70	3.48
hsa-miR-29b-1-5p_L-1	CTGGTTTCATATGGTGGTTTAGA	0	16.38	0	13.24
hsa-miR-31-3p_R+1	TGCTATGCCAACATATTGCCATC	0	0	12.22	17.41
hsa-miR-33b-5p_R-1	GTGCATTGCTGTTGCATTG	0	14.04	136.29	27.17

hsa-miR-34c-3p	AATCACTAACCACACGGCCAGG	0	5.85	0	0
hsa-mir-96-p3	TGTGTCTCTCCGCTCTGAG	0	8.19	0	3.48
hsa-mir-106a-p3	ACTGCAATGTAAGCACTTCT	0	0	0	9.06
hsa-miR-129-5p	CTTTTTGCGGTCTGGGCTTGC	0	4.09	0	0
hsa-miR-139-5p	TCTACAGTGCACGTGTCTCCAGT	0	9.36	21.62	10.45
hsa-miR-146a-3p_L+2R-3	GACCTCTGAAATTCAGTTCTT	0	0	15.04	16.02
hsa-miR-147b-5p	TGGAAACATTTCTGCACAAACT	0	0	9.40	34.13
hsa-miR-147b-3p	GTGTGCGGAAATGCTTCTGCT	0	7.02	59.21	62.69
hsa-miR-150-3p_R-1	CTGGTACAGGCCTGGGGGACA	0	14.04	15.04	9.75
hsa-miR-155-3p	CTCCTACATATTAGCATTAACA	0	0	4.70	4.18
hsa-miR-186-3p_L-2R+1	CCAAAGGTGAATTTTTTGGGA	0	0	0	9.06
hsa-miR-188-5p_R+1	CATCCCTTGCATGGTGGAGGGT	0	0	6.58	0
hsa-miR-190a-3p_L+1	ACTATATATCAAACATATTCCT	0	0	45.12	34.13
hsa-miR-192-3p_R+1	CTGCCAATTCCATAGGTCACAGT	0	0	0	4.18
hsa-miR-194-3p	CCAGTGGGGCTGCTGTTATCTG	0	0	0	6.97
hsa-miR-196b-3p	TCGACAGCACGACACTGCCTTC	0	0	7.52	0
hsa-miR-205-3p_L-1R+1	ATTTCAGTGGAGTGAAGTTCA	0	12.87	12.22	0
hsa-miR-219b-5p	AGATGTCCAGCCACAATTCTCG	0	14.04	0	0
hsa-mir-222-p5	AAGGTGTAGGTACCCTCAAT	0	0	7.52	16.02
hsa-miR-301a-5p	GCTCTGACTTTATTGCACTACT	0	0	0	16.02
hsa-miR-301b-3p	CAGTGCAATGATATTGTCAAAGC	0	0	4.70	0
hsa-miR-338-3p_R+1	TCCAGCATCAGTGATTTTGTTGA	0	0	11.28	79.41
hsa-miR-371b-5p	ACTCAAAAGATGGCGGCACTTT	0	0	0	6.27
hsa-miR-377-3p_R-2	ATCACACAAAGGCAACTTTT	0	0	0	4.88
hsa-miR-378b_R+3_1ss14GT	ACTGGACTTGGAGTCAGAAAGA	0	0	4.70	0
hsa-miR-379-5p	TGGTAGACTATGGAACGTAGG	0	0	0	3.48
hsa-miR-379-3p_1ss22TA	TATGTAACATGGTCCACTAACA	0	7.02	0	0
hsa-miR-382-3p_R+1	AATCATTCACGGACAACACTTT	0	0	0	4.18
hsa-miR-409-3p_L+1	CGAATGTTGCTCGGTGAACCCCT	0	0	0	6.27
hsa-miR-410-3p	AATATAACACAGATGGCCTGT	0	0	0	4.88
hsa-miR-422a_1ss10AG	ACTGGACTTGGGGTCAGAAGGC	0	0	0	3.48
hsa-miR-449c-5p_L-1R+1	AGGCAGTGTATTGCTAGCGGCTGTT	0	0	0	13.24
hsa-miR-450a-1-3p	ATTGGGAACATTTTGCATGTAT	0	0	4.70	4.88
hsa-miR-489-3p_R-1	GTGACATCACATATACGGCAG	0	0	12.22	0
hsa-miR-499a-3p_L+1R-4_1ss5AG	GAACGTCACAGCAAGTCTG	0	7.02	4.70	0
hsa-miR-512-5p_L-1R-1	ACTCAGCCTTGAGGGCACTTT	0	0	9.40	0
hsa-miR-514a-3p	ATTGACACTTCTGTGAGTAGA	0	0	9.87	4.88
hsa-miR-515-3p	GAGTGCCTTCTTTTGGAGCGTT	0	0	0	3.48
hsa-miR-518d-5p_R-1	CTCTAGAGGGAAGCACTTTCT	0	3.01	3.09	0
hsa-miR-520c-3p	AAAGTGCTTCCTTTTAGAGGGT	0	0	15.15	4.18
hsa-miR-518e-3p_R-1	AAAGCGCTTCCCTTCAGAGT	0	11.70	24.44	3.48
hsa-miR-518f-5p_R-1	CTCTAGAGGGAAGCACTTTCT	0	3.01	3.09	0
hsa-miR-520b-3p_R+1	AAAGTGCTTCCTTTTAGAGGGT	0	0	15.15	4.18
hsa-miR-519e-5p_R-3	TTCTCCAAAAGGGAGCACT	0	0	4.70	0

hsa-miR-520a-5p_R-2	CTCCAGAGGGAAGTACTTT	0	10.53	26.32	6.97
hsa-miR-520a-3p_R-2	AAAGTGCTTCCCTTTGGACT	0	16.38	15.98	22.29
hsa-miR-520d-3p_R+1	AAAGTGCTTCTCTTTGGTGGGTT	0	0	6.58	0
hsa-miR-539-3p_L-2R+2	CATACAAGGACAATTTCTTTTT	0	15.21	0	0
hsa-miR-545-5p	TCAGTAAATGTTTATTAGATGA	0	0	0	4.18
hsa-miR-548v	AGCTACAGTTACTTTTGCACCA	0	0	10.34	14.63
hsa-mir-548n-p3	CAATTACTTTTGCACCAACC	0	0.88	0	0
hsa-mir-548p-p5_1ss3TG	AAGTAATTGCAGTTTTTGTC	0	0	0	4.18
hsa-mir-548f-1-p5	TTAGGTTGGTGCAAAAGTAAT	0	0.34	0.47	0
hsa-miR-548b-5p_R-1	AAAAGTAATTGTGGTTTTGGC	0	0	0.67	4.88
hsa-miR-548i_R-2	AAAAGTAATTGCGGATTTTG	0	0	0	1.74
hsa-miR-548ah-5p	AAAAGTGATTGCAGTGTTTG	0	0	4.70	0
hsa-miR-548e-5p_L-1R-1	AAAAGCAATCGCGGTTTTTG	0	8.19	0	9.06
hsa-mir-548f-4-p5	TTAGGTTGGTGCAAAAGTAAT	0	0.34	0.47	0

Table S4: Previous reported microRNAs dysregulated in breast cancer were listed and compared with our miRNA basal abundance data. The inferential expression changes of miRNAs in early breast cancer (BC) are analyzed according to reports and our miRNA sequencing data.

**miRNA name**	**miRNA sequence**	**normalized reads**				**Reported Expression in Breast cancer (BC)**		**Inferential Expression in early BC**
		**cfRNA4**	**cfRNA3**	**cellRNA3**	**cellRNA4**	**Results**	**Opposite Results**	
hsa-let-7e-3p_R-2	CTATACGGCCTCCTAGCTTT	90	109	91	89	upregulated	downregulated	upregulated
hsa-let-7a-5p	TGAGGTAGTAGGTTGTATAGTT	69874	57909	83431	129086	upregulated	downregulated	downregulated
hsa-let-7c-5p	TGAGGTAGTAGGTTGTATGGTT	2273	2555	2570	2421	downregulated		downregulated
hsa-let-7c-3p_R-1	CTGTACAACCTTCTAGCTTTC	7	0	6	0			
hsa-let-7b-5p	TGAGGTAGTAGGTTGTGTGGTT	82701	116853	81208	48587	downregulated		downregulated
hsa-let-7b-3p_R-1_1ss21CT	CTATACAACCTACTGCCTTCT	3748	2485	1170	602			
hsa-miR-10b-5p_R-1	TACCCTGTAGAACCGAATTTGT	183	123	70	88	upregulated		
hsa-miR-16-5p	TAGCAGCACGTAAATATTGGCG	7284	7890	12245	13131	upregulated		
hsa-miR-16-1-3p	CCAGTATTAACTGTGCTGCTGA	0	0	23	69			upregulated
hsa-miR-16-2-3p_L+1R-1	ACCAATATTACTGTGCTGCTTT	221	392	564	694			upregulated
hsa-miR-18a-5p	TAAGGTGCATCTAGTGCAGATAG	82	91	794	714	upregulated	downregulated	upregulated
hsa-miR-18a-3p_R-2	ACTGCCCTAAGTGCTCCTTCT	140	171	49	44			upregulated
hsa-miR-19a-3p	TGTGCAAATCTATGCAAAACTGA	196	291	1394	1409	downregulated		
hsa-miR-20a-5p	TAAAGTGCTTATAGTGCAGGTAG	4964	3870	31801	31944	downregulated		downregulated
hsa-miR-20a-3p	ACTGCATTATGAGCACTTAAAG	17	25	155	114			
hsa-miR-20b-5p	CAAAGTGCTCATAGTGCAGGTAG	0	0	36	54	upregulated		upregulated
hsa-miR-21-5p	TAGCTTATCAGACTGATGTTGA	167598	225677	1216907	773412	upregulated		
hsa-miR-21-3p_R+1	CAACACCAGTCGATGGGCTGTC	123	188	2935	2382			upregulated
hsa-miR-25-5p_R+2	AGGCGGAGACTTGGGCAATTGCT	35	42	28	43	upregulated		upregulated
hsa-miR-25-3p	CATTGCACTTGTCTCGGTCTGA	24937	34050	24624	23471			
hsa-miR-29c-5p_R-1	TGACCGATTTCTCCTGGTGTT	672	542	1325	1091	upregulated		upregulated

hsa-miR-29c-3p_R-1	TAGCACCATTTGAAATCGGTT	2999	5933	15753	7487			
hsa-miR-30a-5p	TGTAAACATCCTCGACTGGAAG	143946	97103	178925	183651	downregulated		downregulated
hsa-miR-30a-3p_1ss22CT	CTTTCAGTCGGATGTTTGCAGT	3262	3163	3579	5699			
hsa-miR-30b-5p	TGTAAACATCCTACACTCAGCT	383303	264659	372314	346935	downregulated		downregulated
hsa-miR-34a-5p_R+1	TGGCAGTGTCTTAGCTGGTTGTT	95	113	1263	1481		upregulated	
hsa-miR-34a-3p_R+1	CAATCAGCAAGTATACTGCCCTT	0	0	0	8	downregulated		upregulated
hsa-miR-34c-5p	AGGCAGTGTAGTTAGCTGATTGC	0	0	6	42			
hsa-miR-34c-3p	AATCACTAACCACACGGCCAGG	0	6	0	0			
hsa-miR-92a-1-5p	AGGTTGGGATCGGTTGCAATGCT	25	66	25	35			upregulated
hsa-miR-92a-3p	TATTGCACTTGTCCCGGCCTGT	71133	52695	36108	24183	downregulated	upregulated	downregulated
hsa-miR-92a-2-5p	GGGTGGGGATTTGTTGCATTAC	7	0	0	0			upregulated
hsa-miR-103a-2-5p	AGCTTCTTTACAGTGCTGCCTTG	36	7	53	43	upregulated		upregulated
hsa-miR-103a-3p	AGCAGCATTGTACAGGGCTATGA	38638	22562	35255	76850			
hsa-miR-122-5p_R-1	TGGAGTGTGACAATGGTGTTT	0	156	0	29	upregulated		upregulated
hsa-miR-125b-5p_R-1	TCCCTGAGACCCTAACTTGTG	29145	29131	18317	8758			
hsa-miR-125b-2-3p_L-2	ACAAGTCAGGCTCTTGGGAC	937	560	444	336	upregulated		upregulated
hsa-miR-125b-1-3p	ACGGGTTAGGCTCTTGGGAGCT	10	25	10	10			upregulated
hsa-miR-126-5p_R+1	CATTATTACTTTTGGTACGCGA	0	0	7	23	downregulated		
hsa-miR-126-3p	TCGTACCGTGAGTAATAATGCG	48	76	70	193		upregulated	upregulated
hsa-miR-133a-3p_L-1R+1	TTGGTCCCCTTCAACCAGCTGT	8	4	7	2	downregulated	upregulated	upregulated
hsa-miR-139-5p	TCTACAGTGCACGTGTCTCCAGT	0	9	22	10	downregulated		
hsa-miR-141-3p_R-1	TAACACTGTCTGGTAAAGATG	45058	66296	119600	65598	downregulated		downregulated
hsa-miR-143-5p_1ss22TA	GGTGCAGTGCTGCATCTCTGGA	39	50	207	151	downregulated		
hsa-miR-143-3p_R+1	TGAGATGAAGCACTGTAGCTCT	2268	4251	12322	18655			downregulated
hsa-miR-145-5p	GTCCAGTTTTCCCAGGAATCCCT	138	168	112	367	upregulated		upregulated
hsa-miR-145-3p	GGATTCCTGGAAATACTGTTCT	8	22	58	103			upregulated
hsa-miR-148a-3p	TCAGTGCACTACAGAACTTTGT	386987	588485	918411	876058	downregulated		downregulated
hsa-miR-148b-5p_L+1R-1	GAAGTTCTGTTATACACTCAGG	870	649	598	1076	upregulated		upregulated
hsa-miR-148b-3p_R-1	TCAGTGCATCACAGAACTTTG	20149	21809	40891	61755		downregulated	downregulated
hsa-miR-155-5p_R-1	TTAATGCTAATCGTGATAGGGGT	39	81	1697	1390	upregulated		upregulated
hsa-miR-155-3p	CTCCTACATATTAGCATTAACA	0	0	5	4			upregulated
hsa-miR-181a-5p_R-1	AACATTCAACGCTGTCGGTGAG	24468	26168	17747	14616	downregulated	upregulated	downregulated
hsa-miR-182-5p_R-2	TTTGGCAATGGTAGAACTCACA	29344	16727	23348	40420	upregulated		
hsa-miR-182-3p_L+2R-1	GGTGGTTCTAGACTTGCCAACT	62	28	76	79			upregulated
hsa-miR-191-5p_R-1	CAACGGAATCCCAAAAGCAGCT	221594	117554	51911	87488	upregulated		
hsa-miR-191-3p_R-3	GCTGCGCTTGGATTTCGTC	30	14	7	0			upregulated
hsa-miR-192-5p	CTGACCTATGAATTGACAGCC	671	503	1714	2494	upregulated		upregulated
hsa-miR-192-3p_R+1	CTGCCAATTCCATAGGTCACAGT	0	0	0	4			upregulated
hsa-miR-195-5p_R+1	TAGCAGCACAGAAATATTGGCA	1000	774	3561	1953	upregulated		upregulated
hsa-miR-195-3p_R-2	CCAATATTGGCTGTGCTGCT	50	42	43	40			upregulated
hsa-miR-199a-5p_R-1	CCCAGTGTTCAGACTACCTGTT	22	23	20	19	upregulated		upregulated
hsa-miR-199a-3p_R-1	ACAGTAGTCTGCACATTGGTT	642	716	717	1711			upregulated
hsa-miR-200c-5p	CGTCTTACCCAGCAGTGTTTGG	18	6	65	25	upregulated	downregulated	upregulated
hsa-miR-200c-3p	TAATACTGCCGGGTAATGATGGA	764438	473643	254207	253470			downregulated

hsa-miR-200b-5p	CATCTTACTGGGCAGCATTGGA	5836	6310	2957	2449	upregulated	downregulated	upregulated
hsa-miR-200b-3p	TAATACTGCCTGGTAATGATGA	354432	251845	266377	258231			downregulated
hsa-miR-200a-5p	CATCTTACCGGACAGTGCTGGA	2598	1846	1627	2221	upregulated	downregulated	upregulated
hsa-miR-200a-3p_R+1	TAACACTGTCTGGTAACGATGTT	345502	350091	385973	393857			downregulated
hsa-miR-202		0	0	0	0	upregulated		upregulated
hsa-miR-203a-3p	GTGAAATGTTTAGGACCACTAG	797	2044	3199	1505	upregulated		upregulated
hsa-miR-205-5p	TCCTTCATTCCACCGGAGTCTG	15626	21105	19760	8789	downregulated		downregulated
hsa-miR-205-3p_L-1R+1	ATTTCAGTGGAGTGAAGTTCA	0	13	12	0			
hsa-miR-210-5p_R-1	AGCCCCTGCCCACCGCACACT	58	132	34	20	upregulated		upregulated
hsa-miR-210-3p	CTGTGCGTGTGACAGCGGCTGA	515	1797	4409	1792			upregulated
hsa-miR-215-5p_R+1	ATGACCTATGAATTGACAGACA	0	0	18	26	upregulated		upregulated
hsa-mir-222-p5	AAGGTGTAGGTACCCTCAAT	0	0	8	16	upregulated		upregulated
hsa-miR-222-3p_R+2	AGCTACATCTGGCTACTGGGTCT	679	783	2627	2580			upregulated
hsa-miR-299		0	0	0	0	upregulated		upregulated
hsa-miR-320d_R-1	AAAAGCTGGGTTGAGAGG	186	139	62	18	upregulated		upregulated
hsa-miR-320b_R-2	AAAAGCTGGGTTGAGAGGGC	1206	817	276	252			upregulated
hsa-miR-324-5p_R-1	CGCATCCCCTAGGGCATTGGT	274	370	614	783	upregulated		upregulated
hsa-miR-324-3p_L-3R+1	ACTGCCCCAGGTGCTGCTGGT	313	881	288	182			upregulated
hsa-miR-335-5p_R-2	TCAAGAGCAATAACGAAAAAT	27462	21489	35648	31513	downregulated		downregulated
hsa-miR-342-5p_R+1	AGGGGTGCTATCTGTGATTGAG	228	204	114	54	downregulated		
hsa-miR-342-3p	TCTCACACAGAAATCGCACCCGT	6948	7060	4489	5121			downregulated
hsa-miR-365b-5p_L+1R-2	GAGGGACTTTCAGGGGCAGCT	33	274	5	0			
hsa-miR-365b-3p	TAATGCCCCTAAAAATCCTTAT	12813	10465	6151	8229	downregulated		downregulated
hsa-miR-365a-5p_R-3	AGGGACTTTTGGGGGCAGAT	36	118	0	14			
hsa-miR-373		0	0	0	0	upregulated		upregulated
hsa-miR-375-5p	GCGACGAGCCCCTCGCACAAACC	19	12	0	0	upregulated	downregulated	upregulated
hsa-miR-375-3p	TTTGTTCGTTCGGCTCGCGTGA	170762	305772	53533	42830			downregulated
hsa-miR-376c		0	0	0	0	upregulated		upregulated
hsa-miR-382-5p	GAAGTTGTTCGTGGTGGATTCG	17	0	0	10	upregulated		upregulated
hsa-miR-382-3p_R+1	AATCATTCACGGACAACACTTT	0	0	0	4			upregulated
hsa-miR-409-5p_R-1	AGGTTACCCGAGCAACTTTGCA	14	0	0	23	upregulated		upregulated
hsa-miR-409-3p_L+1	CGAATGTTGCTCGGTGAACCCCT	0	0	0	6			upregulated
hsa-miR-425-5p	AATGACACGATCACTCCCGTTGA	7590	6681	2323	3182	upregulated		
hsa-miR-425-3p_L+1R-1	CATCGGGAATGTCGTGTCCGCC	1157	1007	629	752			
hsa-miR-451a_R-1	AAACCGTTACCATTACTGAGT	4331	6033	3030	806	upregulated		
hsa-miR-497-5p	CAGCAGCACACTGTGGTTTGT	170	598	1645	879	downregulated		
hsa-miR-523-3p_L-1R-2	AACGCGCTTCCCTATAGAGG	11	19	8	0	upregulated		upregulated
hsa-miR-526b-5p_R-1	CTCTTGAGGGAAGCACTTTCTG	7	25	32	14	upregulated		upregulated
hsa-miR-625-5p_R-1	AGGGGGAAAGTTCTATAGTC	7	0	15	12	upregulated		upregulated
hsa-miR-625-3p	GACTATAGAACTTTCCCCCTCA	162	58	46	28			upregulated
hsa-miR-628-5p_R-1	ATGCTGACATATTTACTAGAG	26	48	233	223	upregulated		upregulated
hsa-miR-628-3p_R+1	TCTAGTAAGAGTGGCAGTCGAA	137	171	106	112			upregulated
hsa-miR-652-3p_R+1	AATGGCGCCACTAGGGTTGTGA	6220	4833	2953	3025	downregulated		
hsa-miR-744-5p	TGCGGGGCTAGGGCTAACAGCA	820	462	480	1050	downregulated		

hsa-miR-744-3p_R-1_1ss21CT	CTGTTGCCACTAACCTCAACT	15	14	6	0		
hsa-miR-801		0	0	0	0	upregulated	upregulated
hsa-miR-1287-5p_R-2	TGCTGGATCAGTGGTTCGAG	203	455	418	260	downregulated	
hsa-miR-1287-3p	CTCTAGCCACAGATGCAGTGAT	0	16	5	0		

References

1. Martin M: **Cutadapt removes adapter sequences from high-throughput sequencing reads**. *2011* 2011, **17**(1):3.
2. Chen H, Smith AD, Chen T: **WALT: fast and accurate read mapping for bisulfite sequencing**. *Bioinformatics (Oxford, England)* 2016, **32**(22):3507-3509.
3. Li H, Handsaker B, Wysoker A, Fennell T, Ruan J, Homer N, Marth G, Abecasis G, Durbin R: **The Sequence Alignment/Map format and SAMtools**. *Bioinformatics (Oxford, England)* 2009, **25**(16):2078-2079.
4. Song Q, Decato B, Hong EE, Zhou M, Fang F, Qu J, Garvin T, Kessler M, Zhou J, Smith AD: **A reference methylome database and analysis pipeline to facilitate integrative and comparative epigenomics**. *PloS one* 2013, **8**(12):e81148.
5. Akalin A, Kormaksson M, Li S, Garrett-Bakelman FE, Figueroa ME, Melnick A, Mason CE: **methylKit: a comprehensive R package for the analysis of genome-wide DNA methylation profiles**. *Genome biology* 2012, **13**(10):R87.
6. Chiu RW, Akolekar R, Zheng YW, Leung TY, Sun H, Chan KC, Lun FM, Go AT, Lau ET, To WW *et al*: **Non-invasive prenatal assessment of trisomy 21 by multiplexed maternal plasma DNA sequencing: large scale validity study**. *BMJ (Clinical research ed)* 2011, **342**:c7401.
7. Yoon S, Xuan Z, Makarov V, Ye K, Sebat J: **Sensitive and accurate detection of copy number variants using read depth of coverage**. *Genome research* 2009, **19**(9):1586-1592.
8. Ginestet C: **ggplot2: Elegant Graphics for Data Analysis by H. Wickham**. *Journal of the Royal Statistical Society* 2011, **174**(1):245-246.
9. Nepomnyashchaya YN, Artemov AV, Roumiantsev SA, Roumyantsev AG, Zhavoronkov A: **Non-invasive prenatal diagnostics of aneuploidy using next-generation DNA sequencing technologies, and clinical considerations**. *Clinical chemistry and laboratory medicine* 2013, **51**(6):1141-1154.
